# Impact of a mineral enriched, fiber complex on glycaemic response and satiation in healthy adults: a double-blind, crossover intervention study

**DOI:** 10.1007/s00394-025-03732-8

**Published:** 2025-06-09

**Authors:** Oana Ancu, Richard W. A. Mackenzie, Michael Patterson, Evone Dsilva, Gleb E. Yakubov, Michael Haynes, Sofia Kolida, Christopher G. Stephenson, Adele Costabile

**Affiliations:** 1 School of Life and Health Sciences, University of Roehampton, London, SW15 4 JD UK; 2 Institute of Cardio-Metabolic Medicine, Coventry University, University Hospital Coventry & Warwick NHS Trust, Coventry, CV15 FB UK; 3 Division of Food, Nutrition and Dietetics, School of Biosciences, University of Nottingham, Sutton Bonington Campus, Loughborough, LE12 5RD UK; 4 OptiBiotix Health Plc, Innovation Centre, Innovation Way, Heslington, York Y010 5DG UK

**Keywords:** Appetite regulation, Insulin response, Weight management, Dietary supplements

## Abstract

**Purpose:**

To investigate the effects of a chromium-enriched glucomannan-fructooligosaccharide complex (SB) on glycaemic and insulin responses, satiation, and hunger biomarkers in healthy adults.

**Methods:**

Using a double-blind, placebo-controlled, randomised crossover design, we assessed the acute impact of a single 3 g SB dose in 16 healthy adults (BMI 18.5–24.9 kg/m^2^) during a modified oral glucose tolerance test. On separate days, participants consumed 50 g dextrose or 50 g dextrose with 3 g SB (SBD). Blood glucose and insulin were analysed over 2.5 h. Hunger, fullness, and desire to eat were assessed via visual analogue scales. Additionally, the impact of SB on gastric viscosity was assessed in vitro.

**Results:**

SBD intake significantly reduced the insulin concentration compared to dextrose alone at 45, 75, and 90 min post-intake. Additionally, SBD resulted in significantly greater fullness and a lower desire to eat at 75 min when compared to dextrose (p < 0.05). Although hunger increased over time for both interventions, SBD led to lower hunger, desire to eat, and food desire scores compared to dextrose at 150 min (p < 0.05). The viscosity of SB, even when combined with dextrose, was significantly higher compared to dextrose alone.

**Conclusions:**

These novel findings suggest that SB can modulate insulin response and influence appetite regulation, highlighting its potential use in weight management strategies.

**Supplementary Information:**

The online version contains supplementary material available at 10.1007/s00394-025-03732-8.

## Introduction

Overweight and obesity, characterised by excessive fat deposits and a body mass index (BMI) of 25.0–29.9 and ≥30 respectively, as well as fat mass over 25%, have become a pressing global health concern with far-reaching implications for public health [1]. Excess fat accumulation is now recognised as a major risk factor for numerous metabolic conditions, including type 2 diabetes mellitus, cardiovascular diseases, and certain cancers [2, 3]. In addition to the significant burden on individuals’ health, overweight and obesity pose substantial economic challenges to healthcare systems worldwide [4]. Two recent US population-based studies looking at trends in weight change patterns across the life course, reported that younger adults (25–40 years) gain more weight than middle aged ones, and middle-aged adults gain more than older individuals [5, 6]. Weight gain in early adulthood has been associated with a higher risk of diabetes CVD and CVD mortality, compared to weight gain later in life, further highlighting the need for support in managing weight throughout life. These findings underscore the need for early intervention to support weight management and mitigate long-term health risks, hence our study focused on young adults as the target population. The root causes of weight gain are intricate, involving a complex interplay of genetic predisposition, metabolic dysregulation, behavioural patterns, and environmental factors [7].

Efforts to support a healthy weight throughout life are challenging and the multifactorial nature of weight gain demands a comprehensive and tailored approach to management. Recently, pharma based anti-obesity strategies, have been revolutionised by the application of Glucagon-Like-Peptide 1 (GLP-1) and Glucose-Dependent Insulinotropic Polypeptide (GIP) receptor agonists. GLP-1 receptor agonists delay stomach emptying, which stimulates the feelings of fullness and suppresses appetite, facilitating reduced food intake and effective weight loss. Additionally, dual GLP-1/GIP receptor agonists enhance insulin secretion in a glucose-dependent manner, further improving glycaemic control. However, these interventions often come with undesirable side effects, with the most common and least severe including nausea, constipation, diarrhoea, vomiting and abdominal pain.

Given the limitations, risks and suitability of pharma-based approaches, functional foods and supplements may offer a beneficial alternative. Strategies which support the regulation of appetite may help control daily energy intake and contribute to effective body weight management. Approaches that effectively address glycaemic response, metabolic responses, and satiation, whilst mitigating potential adverse effects are of high interest and have been reported as effective means of improving body weight management [8–10]. A mineral enriched, prebiotic-fibre complex, composed of glucomannan, fructooligosaccharides (FOS) and chromium (III), has been shown to support sustainable weight management and promote gut microbiome diversity, through reducing hunger, fat intake and food cravings in human intervention studies [11–13].

The present study aims to investigate the acute impact of a single dose (3 g) of chromium (III) enriched glucomannan-FOS complex intake, on the glycaemic and insulin response during the oral glucose tolerance test (OGTT). The primary outcome is the change in postprandial insulin and glucose level following the intervention. Additionally, as secondary outcomes, this study aims to elucidate the potential benefits in managing postprandial glucose fluctuations and how these translate in the perception of fullness, hunger, and desire to eat. These subjective responses play a vital role in individuals’ dietary choices and overall eating behavior. Understanding how this dietary supplement may influence subjective satiety measures can shed light on its potential as a tool for appetite management and weight control.

The in vitro impact of the supplement on the evolution of viscosity in simulated gastric conditions was also investigated and correlated with the in vivo findings.

## Methods

### Ethical approval and study design

A double-blinded, placebo-controlled crossover intervention trial was conducted at the University of Roehampton, School of Health and Life Sciences, UK to assess the effects of a mineral enriched, prebiotic-fibre complex (SlimBiome®; SB: glucomannan, fructooligosaccharides-FOS and chromium), on glucose control and satiation in healthy adults undergoing the modified oral glucose tolerance test (OGTT) [14–16]. Participants were randomly assigned using a computer-generated list, and the investigator was blinded, as drinks were prepared by technical staff, labelled with coded identifiers, and made identical in taste and smell to prevent identification. Ethical approval was granted by the University of Roehampton Research Ethics Committee (reference number LSC 18/238; clinicaltrials.gov ID: NCT04250831). Participants were recruited through advertisements placed locally and online through university wide emails. All experimental procedures were in accordance with the Declaration of Helsinki for Medical Research Involving Humans [17].

### Inclusion/exclusion criteria

Eligibility criteria included: healthy males and females, aged 18–65 years, with a Body Mass Index (BMI) between 18.5–24.9 kg/m^2^, normal fasting glucose concentrations (between 3.9 and 5.6 mmol/l), ability to comply with all study’s procedures and follow nutritional advice and habitually consume three standard meals a day (i.e. breakfast, lunch and dinner). Individuals with a BMI outside of the study’s range (<18.5 kg/m^2^ or >24.9 kg/m^2^); or suffering from any significant health conditions (i.e. neuropathy, nephropathy, retinopathy, vascular diseases, strokes, hypertension, cardiovascular disease, hypercholesterolaemia, diabetes or gastro-intestinal disorders); those with anaemia; those who were pregnant, planning to become pregnant or breastfeeding; or were current smokers and those who have recently (<9 months) ceased smoking; or requiring insulin or any other glycaemic altering medication, were excluded from this study. In addition, individuals who had taken any medication, or supplements known to affect appetite, or weight within the last month or during the study, or have significantly changed their physical activity levels in the past 2–4 weeks, or who intend to change it during the study, were also excluded from participation.

### Participants and preliminary measurements

Sixteen individuals (8 males and 8 females) were recruited in this study. Subjects’ anthropometric characteristics are presented in Table 1. Participants were required to attend the laboratory for a first screening and familiarisation visit that lasted around 1.5 h. The first part of the sessions was used to describe the research study to the participants and gain written informed consent for participation in the study.

**Table 1:** Participants’ baseline characteristics

Gender	Males (n = 8)	Females (n = 8)
Age (y)	26 ± 3.4	25.6 ± 3.2
Weight (kg)	82.3 ± 6.5	66 ± 11.8
Height (m)	1.8 ± 0.1	1.6 ± 0.1
Body mass index (kg/m^2^)	25.2 ± 1.8	24.8 ± 3.3
Waist (cm)	86.3 ± 6.5	78.1 ± 8.8
Hip (cm)	104.1 ± 6.7	100.1 ± 9.2
W:H ratio	0.8 ± 0.1	0.8 ± 0.1
Percentage body fat (%)	21.9 ± 3.5	35.8 ± 7.9
Systolic BP (mm Hg)	118.8 ± 5.1	116.4 ± 7.8
Diastolic BP (mm Hg)	74.6 ± 6.2	78.9 ± 8.6
Resting Heart rate (bpm)	59.3 ± 5.4	66.3 ± 5.7
Fasting glucose (mmol/L)	4.6 ± 0.2	4.6 ± 0.2

Data presented as average ± SD, n = 16 (n = 8 males and n = 8 females)

### Intervention protocol

Volunteers attended the laboratories at the University of Roehampton on three different occasions: visit 1 (familiarisation), visit 2 (dextrose) and visit 3 (dextrose and SB solution- SBD). Visits 2 and 3 were completed in a randomised order, with a washout period of at least 2 days between them [14]. Before attending the laboratories, the participants were instructed to fast for approximately 12 h and refrain from consuming alcohol and large meals prior to the study day. Participants were also advised to avoid rigorous physical activity in the morning of the trial.

The first visit was also used to obtain preliminary measurements such as body fat percentage using bioelectrical impedance analysis (BIA), blood pressure readings, and anthropometric indices such as height, weight, waist and hip circumference, as described by Keleszade and colleagues [12]. These measurements allowed for calculations of BMI by dividing weight by height squared (kg/m^2^) and waist-to-hip ratio. All measurements were taken by the same investigator to ensure consistency in the study.

Participants attended the laboratories for the modified OGTT (visits 2 and 3) at approximately 9:00am. To allow for measurement of fasting (baseline) glucose and insulin concentration, a capillary blood sample was collected (as described in the section below). Volunteers ingested in 2–5 min either the dextrose or dextrose and SB (SBD) solutions, following which, blood samples were taken at 15, 30, 45, 60, 75, 90, 120 and 150 min after the first sip of the drink.

### Blood collection and storage

Briefly one of the fingers was held in an upward position and was lanced on the palm side of the finger with a lancet. The finger was pressed firmly by the researcher when the puncture was made to allow enough blood to be obtained. No pressure was applied on the punctured site to avoid dilution with plasma. The blood was collected into a chilled microvette capillary blood collection tube treated with di K-EDTA anticoagulant (CB 300, K_2_E; Sarstedt Ltd), and then gently inverted 5–10 times to allow mixture with the additive, and prevent blood clots from forming. The microvette tube was placed on ice until the experimental trial was completed (a maximum of 2.5 h). The tubes were then centrifuged at 2000 G for 10 min and the resulting plasma was moved into 1.5 ml Eppendorf tubes that were stored at −80 °C until further analysis.

### Blood and plasma analysis

Blood glucose concentrations were determined using Biosen C-Line (EKF Diagnostics, UK). The analysis and calibration were completed using quality control procedures as per the manufacturer’s recommendations. Plasma insulin concentrations were measured using a commercially available human insulin assay (V-Plex Human Insulin Kit, Meso Scale Diagnostics LLC, USA) as per the manufacturer’s recommendations.

### Subjective measurements of appetite

Participants were asked to complete a questionnaire assessing their hunger, fullness, desire to eat, amount of food that they could have ingested, and thirst at baseline (0 min), 75 min and 150 min post-control drink. Responses were recorded using visual analogue scale (VAS) scores ranging from 0 to 100 mm and were adapted from Dalton and colleagues [18].

### Test drinks and preparation

The drinks for the study were prepared in the food laboratory at the University of Roehampton. The dextrose drink was prepared by diluting 50 g dextrose (Bulk Pure Dextrose, Bulk, UK) into 250 ml water, whereas the SBD drink was prepared by diluting 50 g dextrose and 3 g SB (Glucomannan, 33.33%; FOS, 66.67%; chromium 0.003%) into 250 ml water.

### In vitro analysis of viscosity

The viscosity kinetics of SB, Dextrose and SBD under gastric pH conditions were assessed using a rapid viscoanalyser (RVA), (Perten Instruments, Australia).

Each test sample was prepared in gastric juice in the required proportions to achieve a total volume of 30 ml, reflecting the composition tested in vivo, in the OGTT. The gastric juice consisted of sodium chloride (35 mM), hydrochloric acid (0.08 mmol/l), pig gastric mucin (1% w/v, Type II, Sigma Aldrich), and pepsin from porcine gastric mucosa (0.267% w/v, equivalent to 9325 Units/mL, Sigma Aldrich) [19, 20]. The pH was adjusted to mimic the acidity of the stomach (around 1.5–2.0) through hydrochloric acid (HCl) addition. Subsequently, the gastric pH test samples were mixed with de-ionised water, and 4 ml of the prepared gastric juice was added to each sample.

Following method optimisation to allow for SB’s maximum viscosity to be reached, a 30 min RVA run (Model RVA 4800, Perten Instruments, Australia) was conducted at 37 °C and 160 rpm. Test samples were weighed into dry, canisters followed by the addition of gastric juice. To ensure uniformity and homogeneity, the solution underwent thorough mixing (960 rpm) for a duration of 20 s. Each test sample was tested in duplicate.

## Data and statistical analysis

We estimated a sample size using G*Power [21] based upon the association between the three blood glucose levels assessed with OGTT. This indicated that a sample size of 15 participants undergoing OGTT would be sufficient to detect a large effect size (0.5), with a power of 80% and an alpha significance level of 0.05 [16].

Normally distributed continuous variables are presented as Mean ± Standard Deviation (SD); nonnormally distributed continuous variables are presented as median with interquartile range. Continuous variables were compared between groups using t-test (normally distributed) and Mann–Whitney U test (nonnormally distributed) as appropriate. Values of p < 0.05 were considered statistically significant.

Area under the curve (AUC) for glucose and insulin were calculated using GraphPad Prism 8.3.1 software (GraphPad Software, San Diego, USA) using the trapezoid rule. Early insulin response (insulinogenic index) was calculated as (I_30_ − I_0_)/(G_30_ − G_0_), where I_30_ and G_30_ are insulin and glucose concentrations at min 30 post drink ingestion and I_0_ and G_0_ are insulin and glucose concentrations at min 0 (baseline). Moreover, insulin sensitivity was calculated with Cederholm index using the following formula: (75,000 + (Glucose minute 0 − Glucose minute 120) × 19 × weight)/((AUC Glucose/18) × log (AUC insulin/120)), with weight expressed in kilograms. Matsuda index (insulin sensitivity) was calculated as $$\frac{{10,000}}{{\sqrt {G0*I0*Gmean*Imean} }}$$, where G0 = glucose at 0 min, I0 = insulin at 0 min, Gmean = average of glucose concentration during OGTT, Imean = average of insulin concentration during OGTT.

The Kolmogorov–Smirnov test was performed on all variables to determine whether the data was normally distributed. A repeated measures ANOVA was run to check for differences between conditions (dextrose and SBD) for glucose and insulin over time. A two-sample t-test was run to check for differences in AUC_glu_, AUC_ins_ and early insulin response between the two conditions (dextrose and SBD). Subjective parameters (hunger, fullness, desire to eat, and amount of food) rated on the VAS score were analysed using a within subject repeated measures ANOVA with two conditions. Comparisons were conducted both between the two conditions and longitudinally, relative to each condition’s baseline measurement. However, to minimise the number of statistical comparisons, it was chosen to compare between treatments at the same timepoints as for the VAS scores. In addition, the peak values and area under the curve (AUC) were also compared between groups. The paired t-test was again used for the analyses. All statistical analysis tests were conducted using IBM SPSS Statistics version 28.1.1.1.(15) (IBM Corporations, Chicago, IL). Data is expressed as mean (SEM), unless otherwise specified. Statistical difference was accepted at p < 0.05. The total viscosity data obtained through the RVA in vitro experiments were analysed using the one-tailed t-test to compare SBD, dextrose and SB (Microsoft Excel 2024 v16.8). Statistical significance was considered when p < 0.05.

## Results

In the current study we investigated blood glucose and plasma insulin concentrations at baseline and over a period of 150 min, following test drink intake in 16 healthy individuals as a primary outcome. Subjective measures of appetite were used as secondary outcomes.

**Fig. 1:**
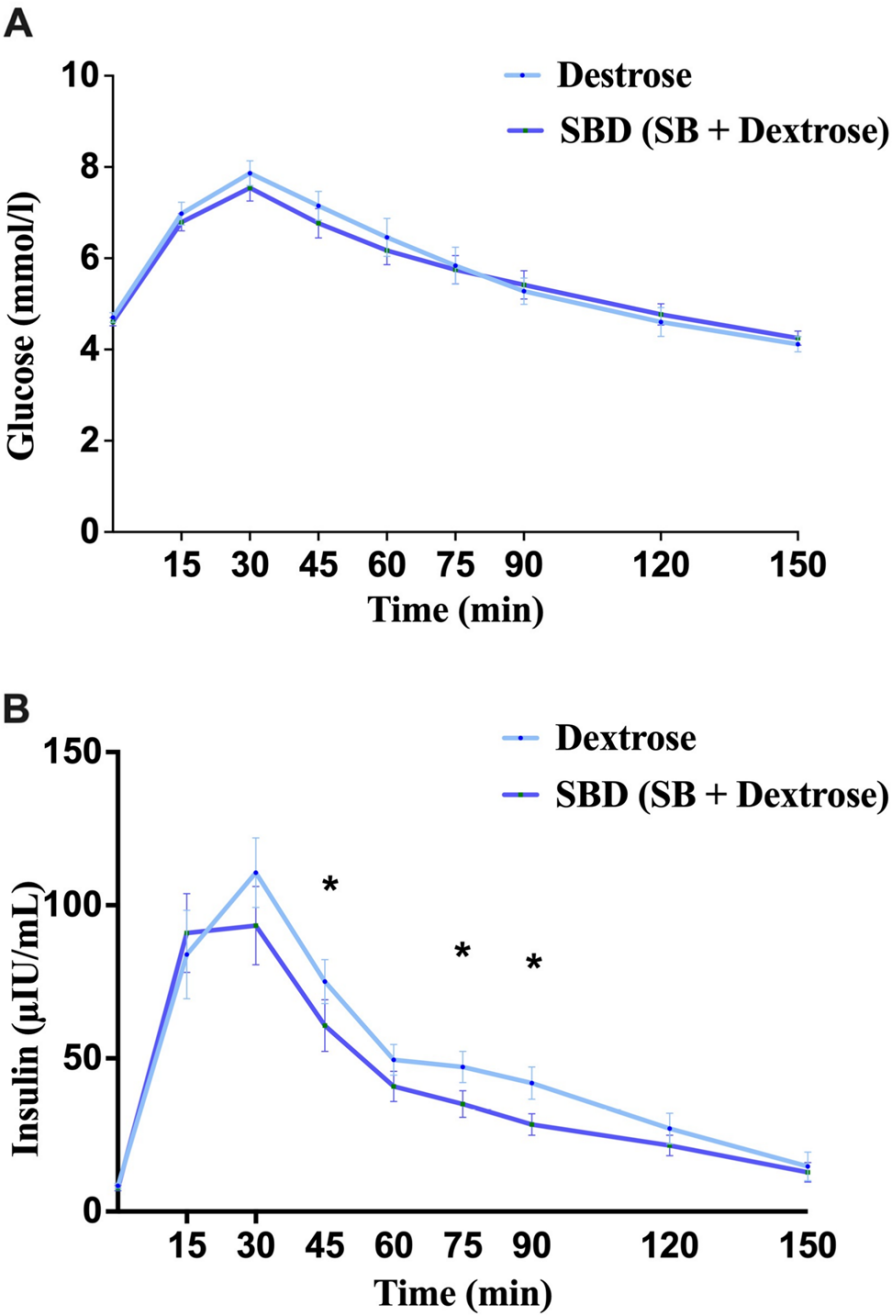
Blood glucose (**A**) and plasma insulin (**B**) concentrations following Dextrose (in light blue) and SBD (SB + dextrose) (navy blue) over time. * denotes statistically significant difference between the two conditions at the specific time point, p < 0.05. Data expressed as Mean ± SEM. SlimBiome®; SB

Blood glucose concentrations did not differ statistically significantly between the two interventions at any time point throughout the OGTT (p > 0.05) (Fig. 1A); however, plasma insulin concentrations were statistically significantly lower with SBD compared to the dextrose trial at 45 min (p = 0.033), 75 min (p = 0.036) and at 90 min (p = 0.017) post-intake (Fig. 1B). A summary of the analysis results is shown in Table 1S.

**Fig. 2:**
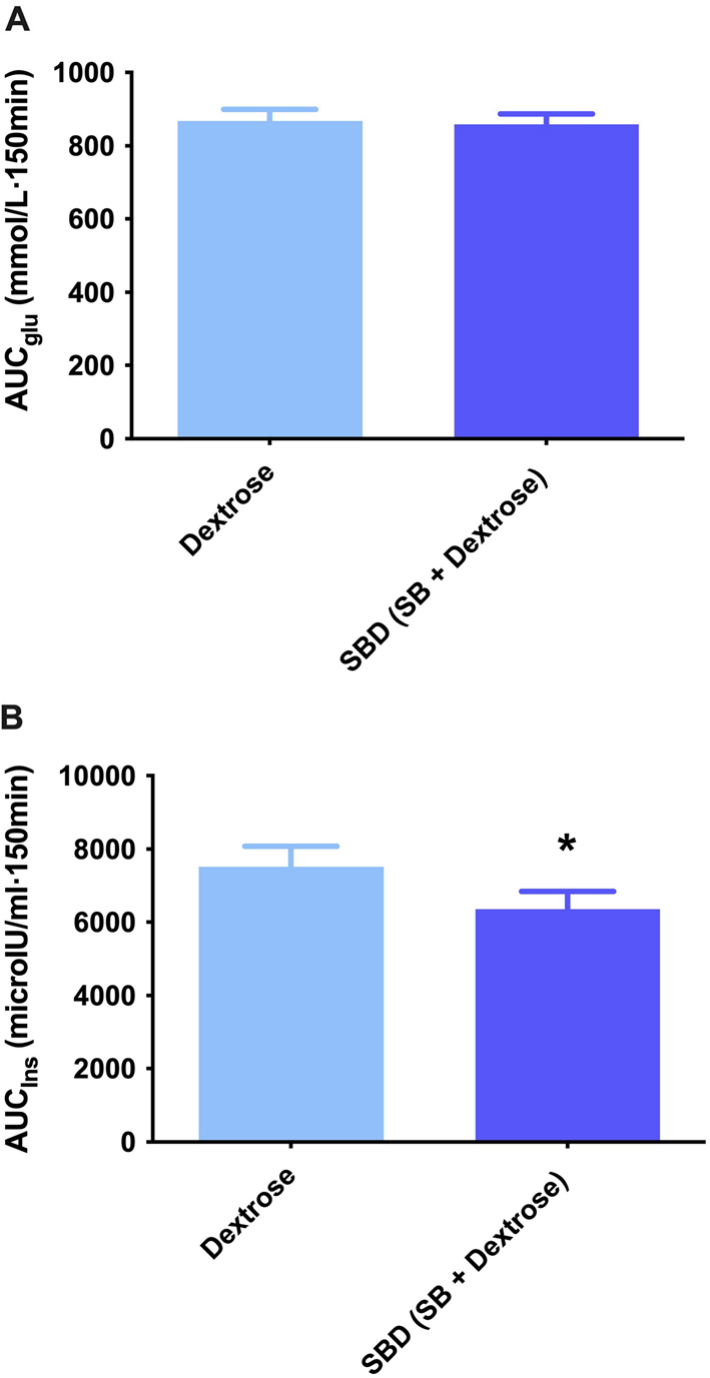
Area under the curve for glucose (**A**) and insulin (**B**) concentrations 150 min post dextrose and SBD ingestion. * Denotes significant difference between the two interventions, p < 0.05. Data expressed as Mean ± SEM. SlimBiome®; SB

**Fig. 3:**
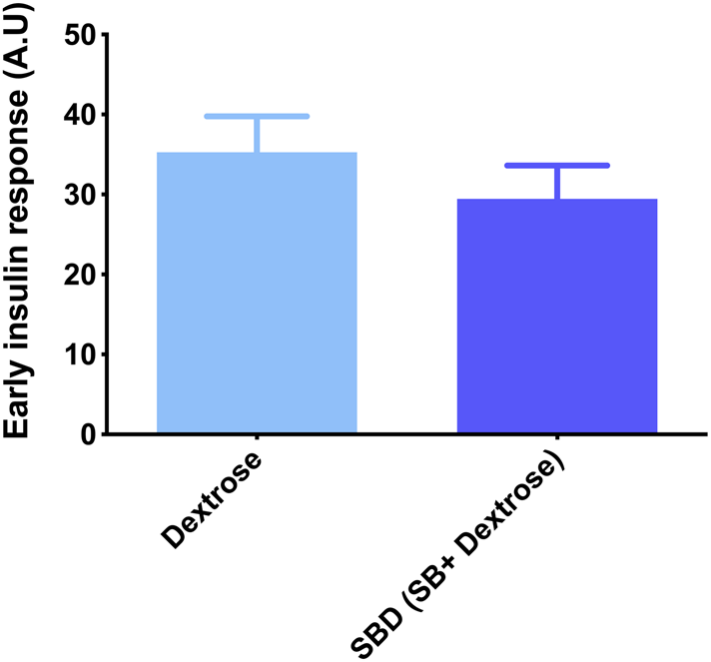
Early insulin response (Insulinogenic index, the initial insulin release within the first 30 min following glucose load) for Dextrose (light blue color) and SBD (SlimBiome®, SB + Dextrose) trial (navy blue colour). Data expressed as Mean ± SEM

The area under the curve for glucose (AUC_glu_) (Fig. 2A) was not significantly different between the interventions (p = 0.731), while area under the curve for insulin (AUC_ins_) **(**Fig. 2B) was significantly lower in the SBD trial when compared to dextrose trial (p = 0.034). Early insulin response (Insulinogenic index) (Fig. 3) was not significantly different between the interventions (p = 0.127).

Plasma insulin was similar in the two groups at baseline. However, at 75 min the levels were significantly lower after receiving SBD. At this timepoint, the mean value was 47 for Dextrose, compared to only 35 for SBD, a mean difference of 12 units. The two treatments also varied significantly in terms of the change from baseline to 75 min, with the increase during this period being lower for SBD. Insulin values at 150 min did not vary between groups, nor did the change in insulin from baseline to 150 min. Peak insulin also did not significantly vary between groups. The final analyses compared changes in both glucose and insulin over time for each of the two treatments separately. A summary of the analysis results is reported in Table 2S.

The results for glucose suggested that at 75 min, values were significantly higher compare to baseline for both treatments. Conversely at 150 min, glucose values were lower than at baseline for both Dextrose and SBD. Insulin values at 75 min were substantially higher than at baseline for both treatments. There was no strong evidence of a difference between insulin values at baseline and at 150 min in either group.

Subjective appetite measures for hunger, fulness, desire to eat and amount of food desired were evaluated at baseline, 75 min, and 150 min post-intake. Baseline VAS scores did not differ significantly between SB and SBD (Table 3S). Evaluating the longitudinal impact on VAS measures, statistically significant increases were observed in hunger (p = 0.001) and the amount of food desired (p = 0.002) between baseline and 150 min upon dextrose intake. SBD intake resulted in statistically significant increases in fullness 75 min post intake (p = 0.012), an effect that was not observed with dextrose (Table 4S).

**Fig. 4:**
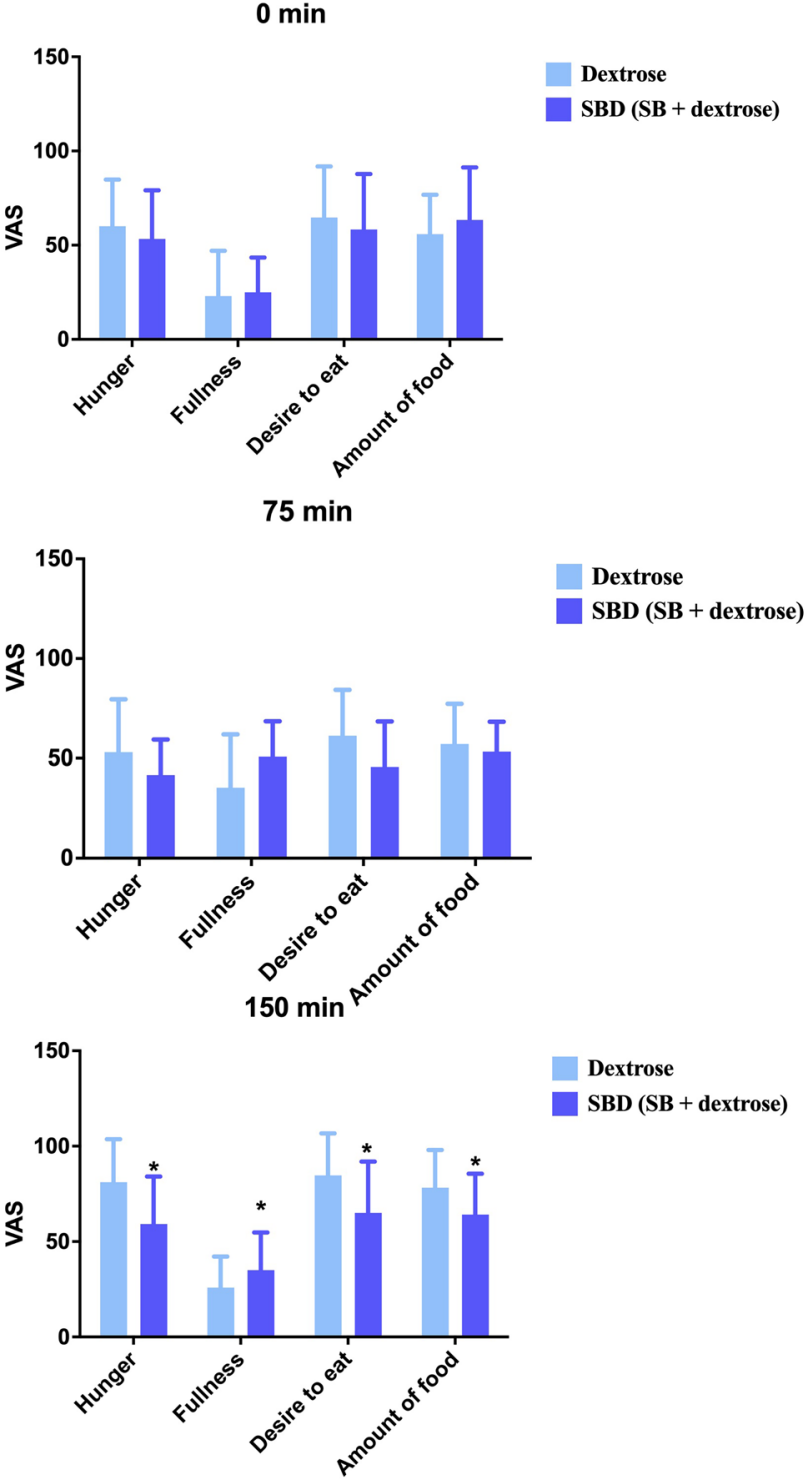
Visual analogue scale (VAS) scores for hunger, fullness, desire to eat, and amount of food measured at baseline (0 min) (**A**), 75 min (**B**) and 150 min (**C**) post intervention drink. The bars in light blue color represent Dextrose and in navy blue SBD (SlimBiome® + Dextrose) trial. * denotes a significant difference between the two interventions, p < 0.05. Data expressed as Mean ± SEM

ANOVA was used on VAS scores (Fig. 4) to evaluate the impact of the treatment for each satiation response. At 150 min post drink ingestion, participants reported feeling significantly less hungry with SBD by 20.7 (−38.0, −3.3; p = 0.038) VAS units, corresponding to a 37.2% difference compared to dextrose. At the same timepoint, participants felt fuller with SBD by 13.3 (5.8, 20.8; p = 0.002) VAS units, corresponding to an increase of 55.4% compared to dextrose. Similarly, desire to eat was lower by 18.7 (−29.5, −7.8; p = 0.002) corresponding to a 32.4% difference compared to dextrose. The amount of food desired was in line with hunger and desire to eat. Participants reported a desire to consume significantly less food by 14.0 (−25.8, −2.2) VAS units with SBD corresponding to a 24.6% difference to dextrose at 150 min. No significant differences were observed between the two interventions at baseline (0 min) and at 75 min post drink ingestion (p > 0.05) (Table 4S**).**

**Fig. 5:**
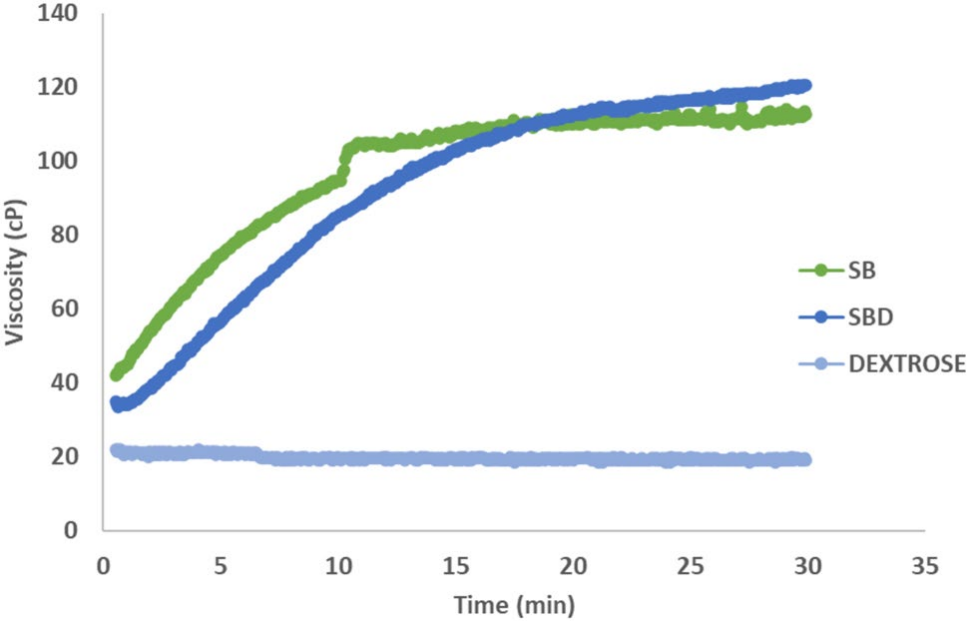
Comparison of the in vitro evolution of viscosity for SB, SBD, and dextrose in gastric pH conditions over 30 min (n = 2), (SlimBiome®: SB, SlimBiome® + Dextrose: SBD)

**Fig. 6:**
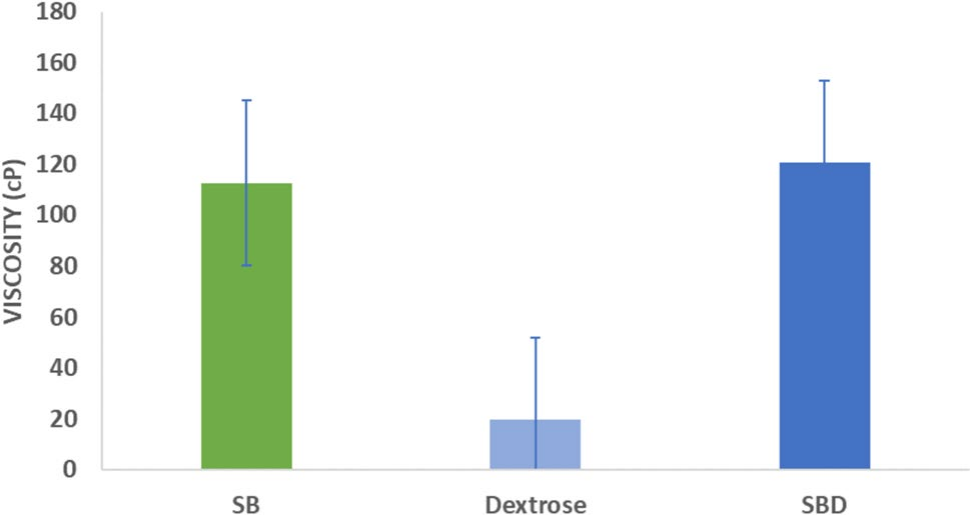
In vitro total viscosity comparison of SB, SBD, and dextrose in gastric pH conditions over 30 min, the error bars represent Mean ± SD (n = 2), (SlimBiome®: SB, SlimBiome® + Dextrose: SBD)

The viscosity of SB, dextrose and SBD were assessed in vitro, under gastric conditions using the 30-min run method with RVA. Considering the kinetics of viscosity development, both SB and SBD reached maximum viscosity between 15 and 20 min, which stabilised thereafter at the experimental conditions (Fig. 5**)**. Figure 6 shows the total viscosity for SB, dextrose and SBD. The viscosity of dextrose was statistically significantly lower compared to both SB (p = 0.01) and SBD (p = 0.03). No significant differences were observed between SB and SBD in the gastric environment (p > 0.05).

## Discussion

The current study aimed to investigate the impact of a chromium enriched, glucomannan-FOS complex, combined with glucose-based drinks on glycaemic response, and its effects on subjective measures of satiety. Specifically, we assessed if the intake of 3 g of SB combined with 50 g dextrose will result in improved glucose and insulin control when compared to ingesting 50 g dextrose alone.

We observed that the intake of the combination of SB and dextrose resulted in a statistically significant decrease in insulin values at 45, 75 and 90 min post drink ingestion, as well as area under the curve for the 150 min postprandially when compared to dextrose alone. This observation highlights the potentially beneficial impact of SB on postprandial insulin response, which is of great importance in the context of metabolic health and glycemic control. Excessive postprandial insulin increases have been associated with insulin resistance, metabolic dysregulation, and an increased risk of developing type 2 diabetes mellitus [22, 23]. The modulation of postprandial insulin response, as seen in this study, could have implications for improved glucose utilization an important element of metabolic disorders. Early insulin response is of high importance as it reflects pancreatic β-cell function and the body’s ability to regulate post-prandial glucose. The lack of difference in early insulin response between the two treatments may be attributed to the absence of insulin resistance in our study population, as healthy individuals typically maintain an efficient early insulin response, which may not show significant alterations in response to acute interventions. [24].

Several mechanisms may explain the observed reduction in postprandial insulin levels with SB co-ingestion. One possible mechanism involves delaying gastric emptying. Ingestion of dietary fibres, such as those present in SB, haven been previously reported to delay gastric transit time [25–28]. By slowing the rate at which dextrose is absorbed into the bloodstream, SB could attenuate the insulin response to the carbohydrate load, leading to the observed reduction in AUC for insulin. This is also supported by previous studies investigating the effects of delayed gastric emptying on postprandial insulin sensitivity and noticed improvements during oral glucose minimal model [29, 30].

Our in vitro findings on the impact of SB combination with dextrose on viscosity, demonstrate that in conditions simulating aspects of the gastric environment, there is a statistically significant increase in viscosity, which reaches maximum levels within 20 min, confirming previous study findings. Glucomannan forms a viscous gel on exposure to an aqueous environment, absorbing up to 50 times its weight in water [30]. Its gelling properties contribute to weight loss promoting effects, by delaying gastric emptying, slowing bowel transit time, and blunting post-prandial surges in insulin and glucose [26, 30]. The impact of FOS intake on satiety and appetite regulating peptides has been explored in multiple human studies [27]. A recent meta-analysis and systematic review has described the potential benefit of enhancing gut microbiome diversity and stimulating the growth of *Bifidobacterium* spp. and SCFA formation on weight loss [31]. Nevertheless, more prominent effects occur at FOS consumption of 7.5 g/day to 15 g/day and an intervention period of 4 weeks, suggesting dose dependent effects.

Moreover, the inclusion of chromium (III) in the SB formulation may also contribute to the observed effects on insulin response. Trivalent chromium has been implicated in glucose metabolism and insulin signaling pathways [32]. In contrast to trivalent chromium, hexavalent chromium is a highly toxic and mutagenic mineral, and as such all chromium-based supplements must be of the highest purity to ensure safety [32–36]. As shown in an extensive systematic review, this essential trace mineral has been suggested to enhance fasting glucose concentrations by −1.4 mmol/l and glycosylated hemoglobin levels in individuals suffering with diabetes [35]. However, most human intervention studies on the impact of chromium intake on glucose and insulin response, fail to demonstrate an impact on healthy individuals at doses that are aligned with the maximum chromium intake suggested by the WHO and EFSA of 250 μg/d [33, 34]. A meta-analysis [37] evaluated the impact of chromium supplementation on post-load glycaemic metabolism and showed no consistent effect among the 14 studies on participants with normal blood glucose tolerance. Two studies that reported efficacy in post prandial glucose metabolism, were relevant to 400–800 μg chromium per day [38] and 250 μg per day [39]. The chromium content of a single SB dose tested here was 15 μg, markedly lower to that used in previous published work. This implies that it is the combined effect with glucomannan and FOS rather than chromium alone driving the observed reduction in AUC for insulin in a healthy weight, normoinsulinaemic population in this study.

The ingestion of dextrose mediated significant increases in hunger and the amount of food desired, effects that were not observed upon the co-ingestion with SB, where no significant differences were noted between baseline and the end of the OGTT. Additionally, fullness with SBD significantly increased at 75 min and remained significantly higher compared to dextrose at 150 min. These findings suggest that SB intake can promote and sustain the feeling of fullness, while blunting hunger over prolonged periods. The observed reduction in hunger sensation following the consumption of SBD is of particular interest in the context of weight management. Hunger is a powerful physiological driver that can lead to increased food consumption, and uncontrolled hunger sensations are often associated with overeating and difficulty in adhering to calorie-restricted diets [12]. The ability of SB to curb hunger and support fullness could potentially aid individuals in adhering to their dietary regimens, promoting a sustained energy deficit necessary for weight loss [12]. Furthermore, the heightened sensation of fullness observed with the intake of SB aligns with the satiety-enhancing properties of the supplement. A greater feeling of satiety post-meal is linked to reduced food intake at subsequent meals and decreased snacking behavior. By promoting feelings of satiety and reducing hunger, SB may help mitigate excessive calorie intake and improve overall glycemic control, potentially leading to favorable effects on metabolic conditions.

Although this was an acute study and does not account for the potential contribution of bacterial fermentation to appetite regulation, it is in line with the findings of the [13] and [12] studies where statistically significant reductions in hunger and cravings for sweet and savory foods have been reported over 4 weeks of SB supplementation. The results of this study report statistically significant improvements in appetite related parameters, including reducing hunger and supporting the feeling of fullness for prolonged periods compared to controls, at a dose of chromium that is no higher than 15 μg, when combined with FOS and glucomannan in the food supplement under evaluation. Previous studies on the long-term intake of the supplement, have demonstrated that when taken at a dose of 3 g, 3 times/day, it significantly improved gut microbiome diversity and specifically the relative abundance of *Bifidobacterium* spp., Christensenella and Bacteroidetes [12]. The increases in *Bifidobacterium* spp. abundance have been previously related with a potential impact on satietogenic gut peptides.

The underlying mechanisms by which the chromium enriched glucomannan-FOS complex investigated in this study exerts its effects on appetite regulation are likely multifaceted. The presence of glucomannan and fructooligosaccharides associated with delayed gastric emptying and increased gut peptide secretion, as explained above, can influence feelings of satiety [40–42].

## Conclusions

In conclusion, the findings of this study provide valuable insights into the effects of the acute impact of a chromium enriched glucomannan-FOS supplement on insulin response and satiety markers, shedding light on its potential as a promising dietary intervention for metabolic health and weight management. Co-ingestion of SB with dextrose resulted in improved glycemic control and in statistically significantly lower sensations of hunger and increases in fullness, suggesting its potential role in appetite regulation and weight management. These subjective measures of appetite are of great importance in influencing dietary behaviors and energy intake, potentially supporting sustained adherence to dietary regimens essential for weight loss. However, further investigations, including longer-term studies and mechanistic research, are warranted to elucidate the precise underlying mechanisms and to ascertain the sustained effects of SB intake on glucose control and satiety. Nonetheless, the current findings contribute to the growing body of evidence supporting the potential of SB as an effective and safe dietary approach to optimize metabolic health and weight management in individuals seeking to improve their overall well-being.

## Supplementary Information

Below is the link to the electronic supplementary material.

Supplementary file1 (DOCX 28 KB)

## Data Availability

Anonymized data in the manuscript, code book and analytic code can be made available upon reasonable request after publication.
