# Supplementary material for: Impact of a mineral enriched, fiber complex on glycaemic response and satiation in healthy adults: a double-blind, crossover intervention study
Source: Eur J Nutr. 2025 Jun 9;64(5):216. doi: 10.1007/s00394-025-03732-8 (PMC12148962; doi:10.1007/s00394-025-03732-8)
Supplement: Supplementary file 1 — Supplementary file1 (DOCX 28 KB) [file 394_2025_3732_MOESM1_ESM.docx]

**Table1S**. Group comparisons for Glucose and Insulin

| **Outcome** | **Timepoint** | **n** | **Dextrose**  **Mean ± SD** | **SBD**  **Mean ± SD** | **Difference ^(+)^**  **Mean (95% CI)** | **p-value** |
| --- | --- | --- | --- | --- | --- | --- |
| Glucose | Baseline | 16 | 4.70 ± 0.43 | 4.61 ± 0.36 | -0.09 (-0.27, 0.09) | 0.29 |
|  | 75 mins | 16 | 5.84 ± 1.63 | 5.75 ± 1.24 | -0.09 (-0.70, 0.53) | 0.77 |
|  | Baseline to 75 mins | 16 | 1.14 ± 1.35 | 1.14 ± 1.25 | 0.01 (-0.66, 0.67) | 0.98 |
|  | 150 mins | 16 | 4.11 ± 0.67 | 4.25 ± 0.62 | 0.13 (-0.18, 0.45) | 0.38 |
|  | Baseline to 150 mins | 16 | -0.59 ± 0.75 | -0.36 ± 0.58 | 0.23 (-0.12, 0.57) | 0.18 |
|  | Peak | 16 | 8.21 ± 1.15 | 7.81 ± 1.10 | -0.40 (-0.94, 0.15) | 0.14 |
|  | AUC | 16 | 868 ± 125 | 859 ± 115 | -10 (-52, 33) | 0.64 |
| Insulin | Baseline | 16 | 7.5 ± 3.7 | 7.6 ± 3.6 | 0.1 (-2.4, 2.7) | 0.91 |
|  | 75 mins | 16 | 47.2 ± 20.5 | 35.1 ± 17.4 | -12.1 (-23.2, -1.0) | **0.04** |
|  | Baseline to 75 mins | 16 | 39.8 ± 19.0 | 27.5 ± 15.7 | -12.2 (-22.2, -2.3) | **0.02** |
|  | 150 mins | 16 | 14.7 ± 18.7 | 12.8 ± 12.8 | -2.0 (-7.6, 3.7) | 0.48 |
|  | Baseline to 150 mins | 16 | 7.2 ± 19.7 | 5.2 ± 10.5 | -2.1 (-8.6, 4.4) | 0.51 |
|  | Peak | 16 | 116.1 ± 42.5 | 113.9 ± 48.9 | -2.1 (-24.3, 20.1) | 0.84 |
|  | AUC | 15 | 7518 ± 2240 | 6351 ± 1936 | -1167 (-2174, -161) | **0.03** |

(+) Difference expressed as score for SBD minus score for Dextrose

**Table 2S.** Within-group comparisons for Glucose and Insulin

| **Outcome** | **Group** | **Timepoint** | **n** | **Baseline**  **Mean ± SD** | **75/150 mins**  **Mean ± SD** | **Change ^(+)^**  **Mean (95% CI)** | **p-value** |
| --- | --- | --- | --- | --- | --- | --- | --- |
|  |  |  |  |  |  |  |  |
| Glucose | Dextrose | 75 mins | 15 | 4.70 ± 0.43 | 5.83 ± 1.63 | 1.14 (0.41, 1.86) | **0.005** |
|  |  | 150 mins | 15 | 4.70 ± 0.43 | 4.11 ± 0.67 | -0.59 (-0.99, -0.19) | **0.007** |
|  |  |  |  |  |  |  |  |
|  | SBD | 75 mins | 15 | 4.61 ± 0.36 | 5.75 ± 1.24 | 1.14 (0.48, 1.81) | **0.002** |
|  |  | 150 mins | 15 | 4.61 ± 0.36 | 4.25 ± 0.62 | -0.36 (-0.67, -0.05) | **0.02** |
|  |  |  |  |  |  |  |  |
| Insulin | Dextrose | 75 mins | 15 | 7.5 ± 3.7 | 47.2 ± 20.5 | 39.8 (29.6, 49.9) | **<0.001** |
|  |  | 150 mins | 15 | 7.5 ± 3.7 | 14.7 ± 18.7 | 7.2 (-3.3, 17.7) | 0.16 |
|  |  |  |  |  |  |  |  |
|  | SBD | 75 mins | 15 | 7.6 ± 3.6 | 35.1 ± 17.4 | 27.5 (19.1, 35.9) | **<0.001** |
|  |  | 150 mins | 15 | 7.6 ± 3.6 | 12.8 ± 12.8 | 5.2 (-0.5, 10.8) | 0.07 |

(+) Change expressed as score for at 75 or 150 mins minus score at Baseline

**Table 3S.** Longitudinal impact on VAS measures

| **VAS outcome** | **Group** | **Timepoint** | **n** | **Baseline**  **Mean ± SD** | **75/150 mins**  **Mean ± SD** | **Change ^(+)^**  **Mean (95% CI)** | **p-value** |
| --- | --- | --- | --- | --- | --- | --- | --- |
|  |  |  |  |  |  |  |  |
| Hunger | Dextrose | 75 mins | 15 | 49.3 ± 28.7 | 54.7 ± 28.8 | 5.3 (-13.1, 23.8) | 0.55 |
|  |  | 150 mins | 15 | 49.3 ± 28.7 | 77.3 ± 20.2 | 28.0 (11.1, 44.9) | **0.003** |
|  |  |  |  |  |  |  |  |
|  | SBD | 75 mins | 15 | 51.3 ± 25.3 | 42.7 ± 16.7 | -8.7 (-21.2, 3.9) | 0.16 |
|  |  | 150 mins | 15 | 51.3 ± 25.3 | 58.7 ± 23.3 | 7.3 (-5.1, 19.8) | 0.23 |
|  |  |  |  |  |  |  |  |
| Fullness | Dextrose | 75 mins | 15 | 24.0 ± 23.2 | 34.7 ± 28.5 | 10.7 (-5.5, 26.8) | 0.18 |
|  |  | 150 mins | 15 | 24.0 ± 23.2 | 16.7 ± 15.0 | -7.3 (-21.9, 7.2) | 0.30 |
|  |  |  |  |  |  |  |  |
|  | SBD | 75 mins | 15 | 24.0 ± 15.0 | 44.4 ± 18.8 | 20.0 (6.0, 34.0) | **0.009** |
|  |  | 150 mins | 15 | 24.0 ± 15.0 | 30.0 ± 20.0 | 6.0 (-5.4, 17.4) | 0.28 |
|  |  |  |  |  |  |  |  |
| Desire to eat | Dextrose | 75 mins | 15 | 60.0 ± 33.4 | 57.3 ± 26.9 | -2.7 (-19.6, 14.3) | 0.74 |
|  |  | 150 mins | 15 | 60.0 ± 33.4 | 80.7 ± 24.0 | 20.7 (-0.5, 41.9) | 0.06 |
|  |  |  |  |  |  |  |  |
|  | SBD | 75 mins | 15 | 55.3 ± 28.7 | 45.3 ± 22.6 | -10.0 (-24.3, 4.3) | 0.16 |
|  |  | 150 mins | 15 | 55.3 ± 28.7 | 62.0 ± 26.2 | 6.7 (-7.2, 20.5) | 0.32 |
|  |  |  |  |  |  |  |  |
| Amount of food | Dextrose | 75 mins | 15 | 53.3 ± 25.3 | 59.3 ± 22.8 | 6.0 (-5.8, 17.8) | 0.29 |
| desired |  | 150 mins | 15 | 53.3 ± 25.3 | 76.0 ± 25.3 | 22.7 (9.0, 36.3) | **0.003** |
|  |  |  |  |  |  |  |  |
|  | SBD | 75 mins | 15 | 60.7 ± 27.4 | 52.7 ± 15.8 | -8.0 (-19.5, 3.5) | 0.16 |
|  |  | 150 mins | 15 | 60.7 ± 27.4 | 62.0 ± 21.4 | 1.3 (-11.7, 14.4) | 0.83 |

(+) Change expressed as score for at 75 or 150 mins minus score at Baseline

**Table 4S.** Group comparisons for VAS scores

| **VAS outcome** | **Timepoint** | **n** | **Dextrose**  **Mean ± SD** | **SBD**  **Mean ± SD** | **Difference ^(+)^**  **Mean (95% CI)** | **p-value** |
| --- | --- | --- | --- | --- | --- | --- |
|  |  |  |  |  |  |  |
| Hunger | Baseline | 15 | 49.3 ± 28.7 | 51.3 ± 23.3 | 2.0 (-15.3, 19.3) | 0.81 |
|  | 75 mins | 15 | 54.7 ± 28.8 | 42.7 ± 17.7 | -12.0 (-28.5, 4.5) | 0.14 |
|  | Baseline to 75 mins | 15 | 5.3 ± 33.4 | -8.7 ± 22.6 | -14.0 (-30.7, 2.7) | 0.09 |
|  | 150 mins | 15 | 77.3 ± 20.2 | 58.7 ± 23.3 | -18.7 (-28,2, -9.1) | **<0.001** |
|  | Baseline to 150 mins | 15 | 28.0 ± 30.5 | 7.3 ± 22.5 | -20.7 (-38.0, -3.3) | **0.02** |
|  |  |  |  |  |  |  |
| Fullness | Baseline | 15 | 24.0 ± 23.2 | 24.0 ± 18.0 | 0.0 (-15.7, 15.7) | 1.00 |
|  | 75 mins | 15 | 34.7 ± 28.5 | 44.0 ± 18.8 | 9.3 (-6.4, 25.1) | 0.22 |
|  | Baseline to 75 mins | 15 | 10.7 ± 29.1 | 20.0 ± 25.4 | 9.3 (-9.4, 28.1) | 0.30 |
|  | 150 mins | 15 | 16.7 ± 15.0 | 30.0 ± 20.0 | 13.3 (5.8, 20.8) | **0.002** |
|  | Baseline to 150 mins | 15 | 6.0 ± 20.6 | -7.3 ± 26.3 | 13.3 (-5.1, 31.8) | 0.14 |
|  |  |  |  |  |  |  |
| Desire to eat | Baseline | 15 | 60.0 ± 33.4 | 55.3 ± 28.8 | -4.7 (-24.9, 15.6) | 0.63 |
|  | 75 mins | 15 | 57.3 ± 26.9 | 45.3 ± 22.6 | -12.0 (-28.5, 4.5) | 0.14 |
|  | Baseline to 75 mins | 15 | -2.7 ± 30.6 | -10.0 ± 25.9 | -7.3 (-24.8, 10.1) | 0.38 |
|  | 150 mins | 15 | 80.6 ± 24.0 | 62.0 ± 26.2 | -18.7 (-29.5, -7.8) | **0.002** |
|  | Baseline to 150 mins | 15 | 20.1 ± 38.3 | 6.7 ± 25.0 | -14.0 (-34.2, 6.2) | 0.15 |
|  |  |  |  |  |  |  |
| Amount of food | Baseline | 15 | 53.3 ± 25.3 | 60.7 ± 27.4 | 7.3 (-8.9, 23.6) | 0.35 |
| desired | 75 mins | 15 | 59.3 ± 22.8 | 52.7 ± 15.8 | -6.7 (-17.5, 4.1) | 0.21 |
|  | Baseline to 75 mins | 15 | 6.0 ± 21.3 | -8.0 ± 20.8 | -14.0 (-30.1, 2.1) | 0.08 |
|  | 150 mins | 15 | 76.0 ± 23.2 | 62.0 ± 21.4 | -14.0 (-25.8, -2.2) | **0.02** |
|  | Baseline to 150 mins | 15 | 22.7 ± 24.6 | 1.3 ± 23.6 | -21.3 (-42.9, 0.2) | 0.05 |

(+) Difference expressed as score for SBD minus score for Dextrose
